# The COVID-19 Pandemic Impact on Away and Home Victories in Soccer and Rugby Union

**DOI:** 10.3389/fspor.2021.695922

**Published:** 2021-10-18

**Authors:** Adrien Sedeaud, Quentin De Larochelambert, Julien Schipman, Jean-Francois Toussaint

**Affiliations:** ^1^EA7329 Institut de Recherche BioMédical et d'Epidémiologie du Sport (IRMES), Paris, France; ^2^Institut national du sport, de l'expertise et de la performance (INSEP), Paris, France; ^3^Centre d'Investigation en Médecine du Sport, Paris, France

**Keywords:** home advantage, soccer (football), rugby union, COVID-19 impact, empty stadium

## Abstract

**Objective:** To measure the impact of restrictions due to COVID on the proportion of matches won at home, away and draw in professional soccer and rugby union.

**Materials and Methods:** Two samples of professional soccer and rugby union matches were collected from 2012–13 to 2020–21 seasons. For soccer, data involved first and second division matches of the England, Spain, Germany, Italy, France, Belgium, Scotland, Greece, Portugal, and Turkey championships. For rugby union, championships concerned are Premiership Rugby, Celtic League, Top 14, and Pro D2. The proportions of home, away wins and draw were calculated and compared. A chi-square test of independence between years and types of result was realized to identify an overall inhomogeneity.

**Results:** The proportion of away matches won between the 2012–13 and 2020–21 seasons increased significantly from 28.5 ± 1.2% to 32.5 ± 1.5% in soccer and from 38.0 ± 3.6% to 42.8 ± 5.0% in rugby union. In Premiership Rugby championship, the victory percentage at home dropped from 55.8 ± 3.1% when tifosi were present to 45.8 ± 12.8% when they were not.

**Conclusion:** The home advantage was drastically reduced in empty stadiums for several European soccer and rugby union professional championships. It vanished in the Premiership Rugby and Celtic League during the 2020–21 season.

## Introduction

On March 11, 2020, the World Health Organization declared a pandemic (World Health Organization, 2021b). It rapidly brought the world to a halt. On March 11, 2021, 117,573,007 cases were confirmed with 2,610,925 reported deaths (World Health Organization, 2021a). Affected countries applied varying levels of responses throughout the year, including societal restrictions and lockdown, both severely impacting societies beyond the health damages directly attributed to COVID-19. Sport was also put on pause, especially elite sport (Beneke and Leithäuser, 2021). A large number of competitions were canceled or postponed, such as the Tokyo Olympic and Paralympic Games (Gallego et al., 2020). Nevertheless, while implementing strict health protocols, professional championships were able to resume their activities (Carmody et al., 2020; Hughes et al., 2020; Yanguas et al., 2020). A study of 1,337 soccer players, staff, and officials demonstrated that overall infection rates were below the national ones (Schumacher et al., 2021). More than half of infected subjects were asymptomatic; the remaining had only mild symptoms with no one requiring hospitalization (Schumacher et al., 2021). Moreover, no evidence of transmission during training or matches have been found (Schumacher et al., 2021). In a rugby league, interactions between SARS-CoV-2 positive players and other players during matches were analyzed to determine the transmission risk (Jones et al., 2021). Based on the detailed player-to-player interaction through video and GPS analysis, 27 of 28 identified increased-risk contacts returned negative. For the other 100 players involved in the matches, in the following 14 days, five returned positive, and 95 returned negative using RT-PCR SARS-CoV-2 tests during routine screening (Jones et al., 2021). Despite frequent interactions between positive players and other players, SARS-CoV-2 transmission was, therefore, limited during matches (Jones et al., 2021). Jones et al. (BJSM, 2021) suggests that new cases in professional rugby reflect the wider community. With strict hygiene measures, professional football and rugby matches could be carried out safely during the pandemic (Meyer et al., 2021).

However, resumption of sports took place in empty stadiums. In such extraordinary conditions, it remained uncertain whether a “home advantage” was still present in a stadium without spectators (Beneke and Leithäuser, 2021). In the previous world (with stadiums full of spectators and no restrictions), the home advantage was defined as “the consistent finding that home teams in sports competitions win over 50% of the games played under a balanced home and away schedule” (Courneya and Carron, 1992). Under balanced home and away competition, as in the soccer and rugby union championships, the evidence that home advantage exists is clear (Pollard, 1986; Courneya and Carron, 1992; Nevill et al., 1996; Nevill and Holder, 1999; Pollard and Pollard, 2005; Thomas et al., 2008). In soccer, the home advantage is also consistently found in French (Dosseville, 2007), English (Nevill et al., 1996; Thomas et al., 2004), Scottish (Nevill et al., 1996), Italian (Stefani, 1983), Spanish (Sánchez et al., 2009), and South American championships (Pollard, 2006). These home advantage effects in soccer vary from 51 to 78% depending on the country and division (Nevill and Holder, 1999; Pollard, 2006). In the UEFA Champions League, a home advantage is also measured (García et al., 2013; Goumas, 2017) and observed on specific performance metrics (Poulter, 2009). Indeed, in this competition, the home team scored more goals, had more shots, had a greater share of possession, and won more corners than the away team (Poulter, 2009). In the rugby union, a significant home advantage of 61% for 120 matches played in the Six Nations tournament between 2000 and 2007 was measured regardless of the team's quality (Thomas et al., 2008). In the Super 12 rugby union championship and Tri-nations (southern hemisphere international competition), a home advantage effect was also observed (Morton, 2006). Evidence from the literature highlight that crowd factors provide the most dominant causes (Nevill and Holder, 1999). Moreover, a large number of studies shows that the home advantage increases with crowd size (Nevill and Holder, 1999). Two mechanisms are hypothesized: first, the crowd is able to raise the performance of the home competitors relative to the away team. Second the crowd is able to influence the referees so that they subconsciously favor the home team (Nevill and Holder, 1999). Some studies underline that the density of supporters and crowd noise may influence the decisions of sports officials (Nevill and Holder, 1999; Goumas, 2014; Myers, 2014). Some authors indeed put forward that the crowd is important but factors such as learning and familiarity factors, travel factors, and rule factors are also principal ones (Courneya and Carron, 1992; Jones, 2013).

In the context of a resumption of professional football and rugby championships, behind locked doors, does the home advantage effect still persist? The aim of the study is to measure the impact of restrictions due to COVID on the proportion of matches won at home, away, and in a draw in professional soccer and rugby unions.

## Methods

### Sample

#### Soccer

All first-division matches of the England, Spain, Germany, Italy, France, Belgium, Scotland, Greece, Portugal, and Turkey championships and the second division of English, French, German, Italian, Scottish, and Spanish championships are found on the website http://www.football-data.co.uk/ from the 2012–2013 season to the current 2020–2021 season (until March 1, 2021). These data represent 3032, 3098, 3163, 3098, 3098, 3098, 3097, 2946, and 2096 matches for the 2012–13, 2013–14, 2014–15, 2015–16, 2016–17, 2017–18, 2018–19, 2019–20, and 2020–21 seasons, respectively, for the first division. Concerning the second division, 2342, 2342, 2342, 2342, 2342, 2342, 2222, 2119, and 1398 matches for the 2012–13, 2013–14, 2014–15, 2015–16, 2016–17, 2017–18, 2018–19, 2019–20, and 2020–21 seasons respectively have been analyzed.

#### Rugby Union

All the results of the matches of the first English (Premiership Rugby), Celtic (Celtic League, Pro12/Pro14 League), and French divisions (Top 14 and Pro D2) have been retrieved from the website http://www.itsrugby.fr/ since the 2012–13 season until the current season 2020–2021 (March 1, 2021). These data represent 446, 446, 446, 446, 446, 386, 389, 301, and 209 matches for the 2012–13, 2013–14, 2014–15, 2015–16, 2016–17, 2017–18, 2018–19, 2019–20, and 2020–21 seasons, respectively, for first division. Concerning the second division, 240, 240, 240, 240, 240, 240, 240, 181, and 167 matches for the 2012–13, 2013–14, 2014–15, 2015–16, 2016–17, 2017–18, 2018–19, 2019–20, and 2020–21 seasons, respectively, have been analyzed.

Division 2 data for rugby union and soccer has been integrated to compare if the different division were impacted in a different way by the resumption of competitions in empty stadiums.

### Data Analysis

#### All Competitions Combined

For rugby and soccer, we calculated the proportion $p_{i}=\;\frac{n_{i}}{N}$ each season as well as the confidence interval $\pm z\sqrt{\frac{p_{i}\left(1-p_{i}\right)}{N}}$, ∀*i* ∈{*home, away, draw*}, *z*: quantile of the normal distribution of order $1-\frac{\alpha }{2}=0.975$, independent of the championship.

We used a chi-square test of independence between years *X* :{2012 − 2013…, 2020 − 2021} and types of result *Y* :{*Home, Away, Draw*} to identify an overall inhomogeneity between the two variables. If positive, at least 1 year or one type of result is greater (or lesser) than the others. A test for residues of Haberman chi-square was performed to identify the inhomogeneities.

#### By Championship

Samples were categorized into two categories:

1. Before the end of the championships for COVID-19
2. After the resumption of the matches.

For each championship, the proportion as well as the confidence interval was calculated for each type of result (home, away, draw). Each championship and each proportion of result, before and after COVID was compared using a *z*-test. Significance thresholds were set at α = {0.1, 0.05, 0.01} (respectively ^*^, ^**^, ^***^ in the results section).

### Research Ethics and Data Security

Data collection was compliant with the General Data Protection Regulations applied in the European Union and approved by the Institutional Ethics Committee.

## Results

### General Trend

**Figure 1:**
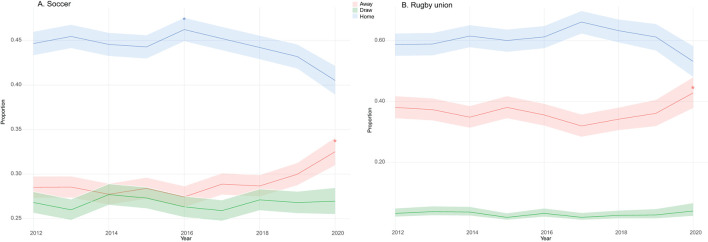
Evolution of the proportion of matches won at home, away, and drawn for the 2012–13 to 2020–21 seasons in **(A)** soccer and **(B)** rugby professional championship.

Figure 1 shows the trend of the proportion of matches won at home, away, and in a draw from the 2012–13 to 2020–21 seasons. For soccer, during the 2012–13 to 2019–20 period, the proportions of matches won at home is fluctuating from 44.7% ± 1.3% in the 2012–13 season to 40.1% ± 1.6% in 2020–21 (Figure 1A). The only significant difference is observed during the 2016–17 season with an increase to 46.2% ± 1.3% match wins at home (Figure 1A). The proportion of matches ending in a draw remains stable from 2012–13 to 2020–21, fluctuating from 26.8% ± 1.1% to 27.0% ± 1.5% (Figure 1A). A significant increase in the proportion of away games won is observed in the 2020–21 season (Figure 1A). The proportion of away matches won between the 2012–13 and 2020–21 seasons increased significantly from 28.5% ± 1.2% to 32.5% ± 1.5% (Figure 1A). For the rugby union, during the 2012–13 to 2019–20 season the proportions of matches won at home remained similar, fluctuating from 58.7% ± 4.0% in the 2012–13 season to 53.2% ± 5.2% in 2020–21 (Figure 1B). The same trend is observed for draws, which fluctuate from 3.8% ± 3.6% in 2012 to 4.0% ± 1.7% in 2020 (Figure 1B). A significant increase in the proportion of away games won is observed in the 2020–21 season (Figure 1B). The proportion of away matches won between the 2012–13 and 2020–21 seasons increased significantly from 38.0% ± 3.6% to 42.8% ± 5.0% (Figure 1A).

### Soccer Championships

**Figure 2:**
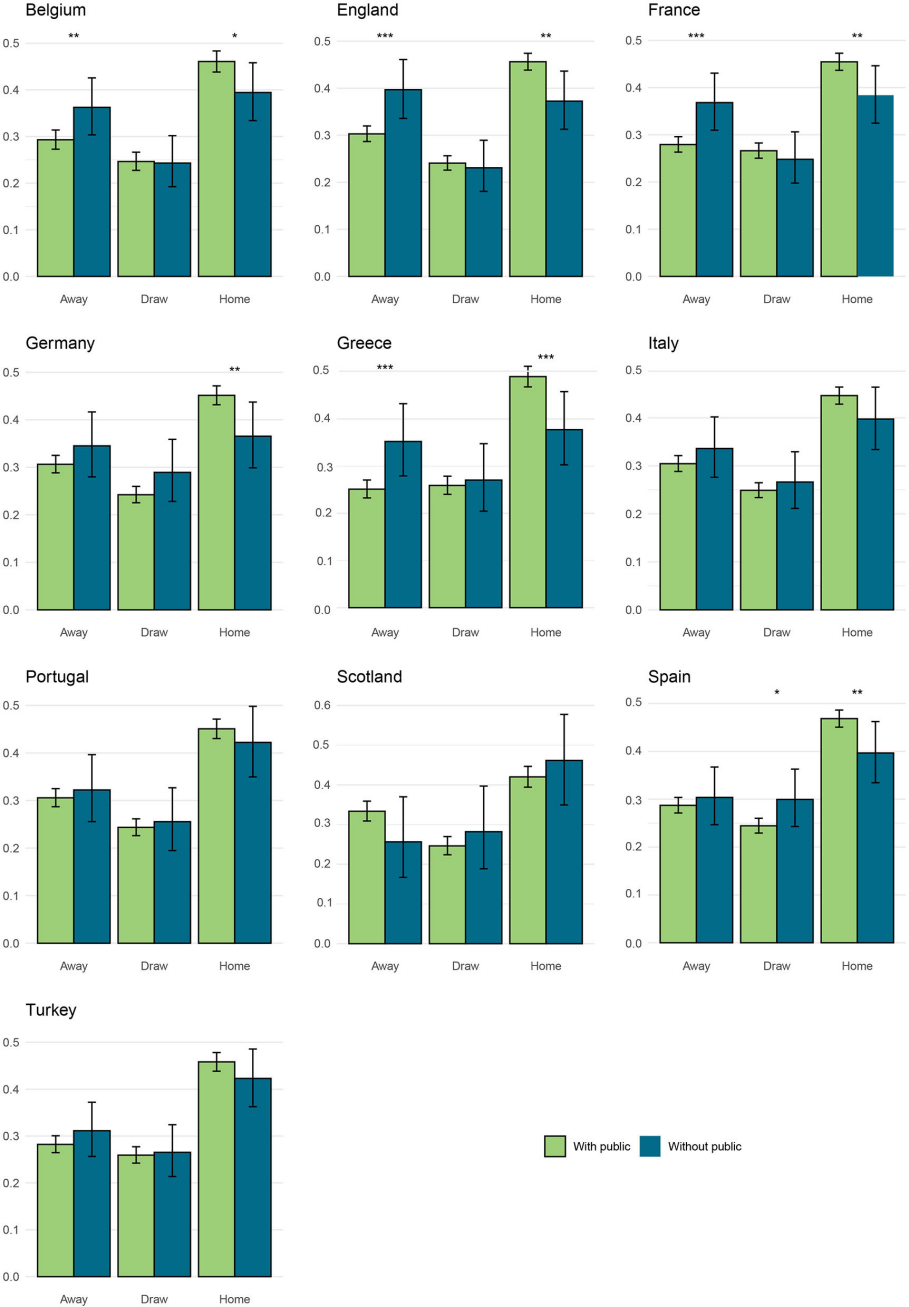
Proportion of matches won at home, away, and drawn in European soccer first division championship with and without supporters. Significance thresholds were set at α = {0.1, 0.05, 0.01} (respectively *, **, ***).

In the French, English, Belgian, and Greek championships, a significant increase in away victories without supporters is observed (Figure 2). For example, in the French championship, a significant increase in away victories from 27.9% ± 1.6% with public in stadium to 36.8% ± 5.8% without supporters is observed (Figure 2). In the English championship, away victory rates increased from 30.3% ± 1.6% with the public in the stadium to 36.7% ± 6.1% without supporters (Figure 2). For Belgium and Greece, the proportion of away matches won has significantly risen from 29.3% ± 2.0% to 25.1% ± 1.9% with public in the stadiums to 36.3% ± 2.9% to 35.2% ± 7.3% without supporters, respectively (Figure 2). In the German championship, the victory percentage at home drops from 45.1% ± 2.0% with supporters to 36.5% ± 6.6% without (Figure 2). A similar trend is observed for Belgium, England, France, Greece, and Spain championships. For the Portugal, Scotland, and Turkey first division championships, no significant differences are observed between the success at home, away, and draws with or without the public (Figure 2). For the second division championship, no significant difference in England, France, and Scotland is measured. For the German second division championship, a significant decrease in draws without the public is observed with a proportion of 29.6% ± 1.8% with the public in the stadiums to 22.3% ± 5.5% without supporters. For the Italian second division championship, a significant decrease is measured in home wins: from 45.6% ± 1.7% with spectators to 35.0% ± 6.0% without the public. In Spain, in the second division championship, a significant increase of away wins occurs between matches with or without spectators. The proportion of matches won away increases from 24.5% ± 1.37% to 29.3% ± 5.1% with the loss of supporters.

### Rugby Union Championships

**Figure 3:**
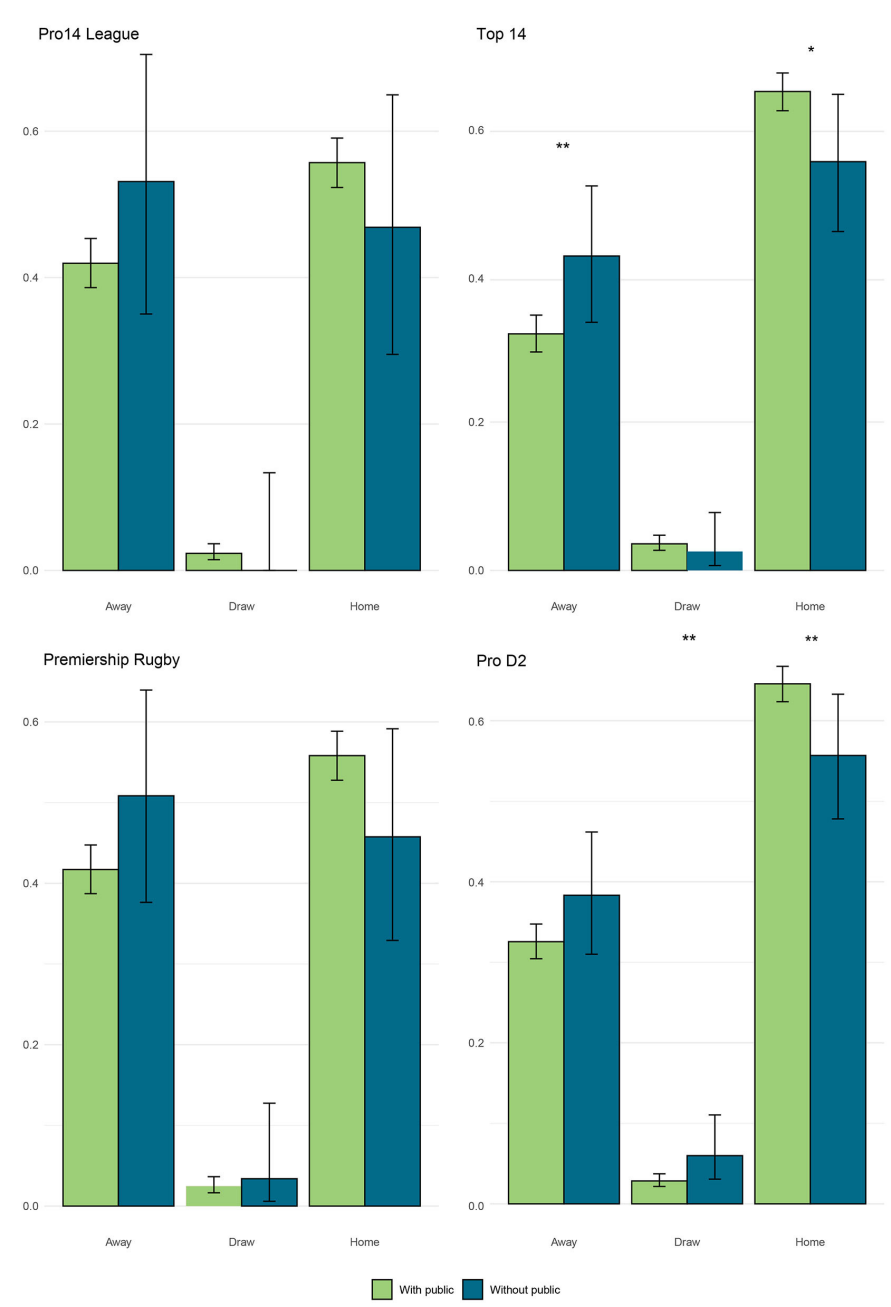
Proportion of matches won at home, away, and drawn in the European rugby union first division championship with and without supporters. Significance thresholds were set at α = {0.1, 0.05, 0.01} (respectively *, **, ***).

In the French Top 14 championship, a significant increase in away victories from 31.9% ± 2.4% with the public in stadium to 42.4% ± 8.9% without supporters is observed (Figure 3). For example, the Stade Rochelais team increases these away wins from 32.0% to 42.9% when matches without supporters resume. In the French Top 14 and Pro D2 championships, playing without spectators resulted in a significant decrease in home wins from 64.5% ± 2.6% to 55.1% ± 9.4% in the top 14 and from 64.6% ± 2.2% to 55.7% ± 7.9% in Pro D2 (Figure 3). For the Premiership Rugby championship, the victory percentage at home drops from 55.8% ± 3.1% with supporters to 45.8% ± 12.8% without a crowd (Figure 3). In this championship, the victory percentage away increases from 41.7% ± 3.0% to 50.8% ± 13.2% (Figure 3). For example, the Bath team sees those home wins drop from 63.6% to 25.1%. For the Pro14 league championship, the victory percentage at home drops from 55.7% ± 3.4% with supporters to 46.9% ± 17.4% without a crowd (Figure 3). In this championship, the victory percentage away increases from 41.9% ± 0.03% to 53.1% ± 18.1% (Figure 3). No significant differences were observed regarding the draw proportion in any league or division.

## Discussion

This study revealed that the effect of the home advantage faded away with the resumption of professional soccer and rugby union championships when no spectators entered stadiums.

### General Trend

The proportion of away matches won in soccer was stable for the 2012–13 to 2018–19 seasons around 28.6%. This proportion of away wins is in accordance with previous studies (Nevill and Holder, 1999; Pollard, 2006). For the 2020–21 season, a significant increase in the proportion of away wins has been measured. The main explanatory hypothesis is that the resumption of championships took place without spectators. Indeed, for some authors, the effects of the home advantage are crowd-mediated (Pollard, 1986). Without the crowd, the possibility to raise the performance of the home competitors and to influence officials are annihilated. The proportion of away matches won in the rugby union was also stable for the 2012–13 to 2018–19 seasons around 36%. In a Six Nations study (Thomas et al., 2008), the home advantage effect ranged from 53% to 70% (mean: 61%), similarly to our results (53.1–66.1%). In the soccer and rugby unions, the proportion of matches won at home remains higher than draws or away matches. A possible explanation lies in other effects of the advantage of playing at home, such as learning and familiarity factors or travel factors (Courneya and Carron, 1992). Indeed, some authors argue that familiarity with local playing conditions was an important factor contributing to home advantage (Schwartz and Barsky, 1977). For example, a study examining the effect of an artificial pitch surface on the home team concludes with a significant advantage for these teams compared with those usually play on traditional grass (Barnett and Hilditch, 1993).

### Soccer Trend

For the English, French, German, Belgian, and Greek championships, playing without supporters leads to an increase in away wins and a concomitant decrease in matches won at home. These results are in accordance with significant decrease of shots (−56.3%) and shots on target (−52.4%) in European top class football matches that were played without spectators due to the COVID-19 pandemic in 2020 (Wunderlich et al., 2021). Once again, the effects mediated by the crowd, familiarity with local playing conditions, and travel factors could be mentioned as explanatory causes (Pollard, 1986; Nevill and Holder, 1999). Other aspects, such as the positive psychological aspect of players performing at home are mentioned (Jurkovac, 1985; Nevill and Holder, 1999). Jurkovac reports feelings of playing better in front of a loud and active crowd at home compared with playing away (Jurkovac, 1985). In this thesis, players declared they were more confident and motivated by visual signs of support, such as banners, when playing in front of a home crowd (Jurkovac, 1985). Familiarity with local playing condition can cause greater confidence with competitors believing they will play better and be more successful at home (Nevill and Holder, 1999). A study by Glamser (1990) assumes that the hostile atmosphere of an away game can produce a dysfunctional aggressive response on the part of the visiting player and a less-than-objective view on the part of officials. Numerous studies observe that officials make more subjective decisions in favor of the home team (Thomas et al., 2006; Lovell et al., 2014). In soccer, Nevill et al. (1996) also highlight that this trend increases in divisions with larger crowds. When these potential aspects are absent, it may reduce the home advantage effect as matches are played without any audience.

### Rugby Union Championships

In the French Top 14, a significant increase in away victories without supporters is observed. Likewise, effects mediated by the crowd on players and officials, and familiarity with local playing conditions are absent when matches are behind closed doors. In Top 14 and Pro D2 championships, playing without spectators resulted in a significant 9% decrease in home wins. The initial home wins were consistent with the 63% observed in Super 12 championship (Morton, 2006). For the Premiership Rugby championship between matches with and without supporters, the victory percentage at home drops by a 10% margin while the away victory increased by 9%. At this time of the 2020–21 season (March 2021), the home advantage no longer exists in Premiership Rugby and Pro14 Leagues.

Although COVID-19 risk factors include sedentarity (De Larochelambert et al., 2020), consequences of the pandemic and subsequent governmental decisions have impacted active populations (Stockwell et al., 2021) and elite athletes with a reduction in well-being, physical capacities, and deconditioning assessed by increased heart rates during lockdown (Pla et al., 2021). Protocols for resumption of championships also influence the competitions themselves. Indeed, this study highlights one of the unanticipated impacts of restrictions caused by COVID-19: the reduction or annihilation of the home advantage in empty stadiums.

### Limitations

The current study has several limitations. First, the performance level of the teams, level differences just before the match, or recent performances were not considered. Second, home advantage is a confounding effect among numerous factors that are operating simultaneously on performance, match outcomes (Nevill and Holder, 1999), and this variable dependency is not integrated. Third, analyses compare the seasons 2012–2019 vs. the 2020–21 season only, producing a lack of data in a situation without supporters; consequently the fluctuations could be due to other non-controlled elements. Fourth, data are based on win, loss, and draw results from websites, which allows highlighting results only on these parameters and not on the others influencing the effects of home advantage. In this study, interplay of different factors as crowd support, referee biases, psychological effects of expectations, travel fatigue, familiarity, territoriality, specific rules, tactical behaviour and competitive balance in a league as covariables are not measured. As mentioned by Wunderlich et al. it seems likely that more than one factor is responsible for the emergence of the home advantage (Wunderlich et al., 2021), and in this specific study only win, loss and draw for a multiple championships have been investigated.

## Conclusion

In England, Spain, Germany, Italy, France, Belgium, Scotland, Greece, Portugal, and Turkey soccer championships, an unanticipated impact of the COVID-19 restrictions is shown with a significant increase in the proportion of away games won during the 2020–21 season. For the rugby union (Top 14, Premiership Rugby, Celtic League, and Pro D2 championships), the proportion of away victories similarly increased. It reveals the reduction of the home advantage in soccer and rugby union professional championships with empty stadiums and its annihilation in the Premiership Rugby and Celtic League in the 2020–21 season.

## Data Availability Statement

The datasets presented in this study can be found in online repositories. The names of the repository/repositories and accession number(s) can be found at: http://www.football-data.co.uk/ and http://www.itsrugby.fr/.

## Author Contributions

QD realized data collection, analyzed the data, and drafted the methods part. AS, JS, and J-FT wrote the manuscript. All authors contributed to the study conception and design, interpretation, provided revisions, and contributed to the final manuscript.

## Conflict of Interest

The authors declare that the research was conducted in the absence of any commercial or financial relationships that could be construed as a potential conflict of interest.

## Publisher's Note

All claims expressed in this article are solely those of the authors and do not necessarily represent those of their affiliated organizations, or those of the publisher, the editors and the reviewers. Any product that may be evaluated in this article, or claim that may be made by its manufacturer, is not guaranteed or endorsed by the publisher.
